# Children’s Environmental Health: A Systems Approach for Anticipating Impacts from Chemicals

**DOI:** 10.3390/ijerph17228337

**Published:** 2020-11-11

**Authors:** Elaine A. Cohen Hubal, David M. Reif, Rachel Slover, Ashley Mullikin, John C. Little

**Affiliations:** 1 US EPA, Center for Public Health and Environmental Assessment, Research Triangle Park, NC 27711, USA; slover.rachel@epa.gov (R.S.); mullikin.ashley@epa.gov (A.M.); 2 Department of Biological Sciences, North Carolina State University, Raleigh, NC 27695, USA; dmreif@ncsu.edu; 3 Department of Civil and Environmental Engineering, Virginia Tech, Blacksburg, VA 24061, USA; jcl@vt.edu

**Keywords:** systems approach, children’s environmental health

## Abstract

Increasing numbers of chemicals are on the market and present in consumer products. Emerging evidence on the relationship between environmental contributions and prevalent diseases suggests associations between early-life exposure to manufactured chemicals and a wide range of children’s health outcomes. Using current assessment methodologies, public health and chemical management decisionmakers face challenges in evaluating and anticipating the potential impacts of exposure to chemicals on children’s health in the broader context of their physical (built and natural) and social environments. Here, we consider a systems approach to address the complexity of children’s environmental health and the role of exposure to chemicals during early life, in the context of nonchemical stressors, on health outcomes. By advancing the tools for integrating this more complex information, the scope of considerations that support chemical management decisions can be extended to include holistic impacts on children’s health.

## 1. Introduction

The volume and total number of chemicals manufactured and used in consumer products has increased dramatically over the last 50 years [1,2]. Organizations around the world are continuously curating lists of chemicals in commerce [3]. The number of chemicals ranges from approximately 23,000 to greater than 68,000 and may include substances that are no longer manufactured but may still occur in the environment. Despite the promise of product and chemical innovations, challenges of evaluating and predicting impacts remain formidable, and only a small number of chemicals have been well studied for environmental health impacts [4,5,6,7]. The gap between the increasing reliance on chemicals in consumer products and our knowledge of the associated human health impacts is growing [8].

Potential health effects from chemicals in products have been associated with the disruption of complex biological processes [9,10]. Increasingly, associations between chemical exposures and the risk of diseases, including asthma, autism spectrum disorder, and childhood obesity, are being reported [11,12,13]. To date, the body of research in this area has been limited, and the complexity of exposures, disease etiology, and health outcomes make it difficult to evaluate and interpret reported associations.

Traditional chemical management strategies focus on evaluating risks from exposures to individual or groups of chemicals based on the properties of those chemicals. However, this approach rarely enables policymakers to link increased risks of specific diseases or public health outcomes to chemical management actions [14]. Community planning and development decisions are designed from the holistic perspective of both minimizing risks and providing an environment that supports and promotes healthy child development. To inform these decisions, information is required on the health impacts of multiple factors in the physical (built and natural) and social environments that contribute to children’s health, as well as the relative importance of each [15].

Gwinn et al. [14] proposed that a public health perspective for chemical risk assessment starts with the disease of concern and incorporates multiple data streams to inform preventative policy decisions. More generally, Lang and Rayner [16] argued that big public health challenges require complex ecological thinking. They advocated for the need to address inherent complexities across four dimensions of existence: Material, biological, cultural, and social. Little et al. [17] proposed a modeling framework that enabled the integration of complex information across multiple social, economic, and environmental systems. The goal of each proposal was to extend the scope of considerations that support robust policy decisions and actions.

To address the full range of public health objectives and ensure sustainable decisions on chemicals, novel approaches are required to incorporate critical elements of complexity and to compare alternatives and evaluate outcomes. Here, we propose a systems approach to enable the integration of information from disparate disciplines to support the holistic consideration of interactions that determine health impacts from early-life exposure to chemicals. Specifically, we conceptually link the chemical source-to-dose framework with the adverse-outcome-pathway (AOP) framework and demonstrate how these frameworks fit in a system-of-systems context to enable further connections to nonchemical stressors. The goal was to extend the scope of considerations that support chemical management decisions to include holistic consideration of children’s health and advance the tools for integrating this more complex information.

## 2. The Complex Problem

### 2.1. Global Burden of Environmental Disease

The World Health Organization (WHO) estimates that, in children under 5 years, 25% of all childhood illnesses throughout the world are due to modifiable environmental factors. Specifically, the WHO estimates considered environmental factors which are realistically amenable to change using available technologies, policies, and preventive public health measures, including contaminated air, soil, and water. However, emerging threats caused by chronic low-dose exposure to chemicals in consumer products were not considered [18]. A WHO analysis focused on public health impacts of chemicals noted that, due to the complexity of evaluating exposures to chemicals, information on the associated disease burden is very limited [19]. In the results of this analysis, WHO reported that, in 2012, at least 1.3 million deaths were attributable to a small number of chemicals for which there were data. In 2016, that number rose to 1.6 million, again only for a small number of chemicals. The report emphasized that people were exposed to many more chemicals in their daily lives. In addition, the United Nations reported that the global goal to minimize adverse impacts of chemicals by 2020 was not achieved [20].

### 2.2. Children’s Environmental Health in the United States

Recent and emerging research findings on the relationship between environmental contributions and children’s health outcomes suggest associations between early-life exposure to environmental contaminants and a wide range of children’s health outcomes, including adverse birth outcomes, asthma, neurodevelopmental disorders, and metabolic disease [21].

Adverse birth outcomes are one of the leading causes of infant mortality and may result in long-term impacts, including motor, cognitive, visual, hearing, and behavioral problems. Birth defects occur in approximately 3% of the births in the United States [22]. These birth outcomes have been associated with exposure to a variety of environmental contaminants in utero and early in life, including fine particulate matter [23,24,25] and chemicals such as arsenic [26], organochlorine pesticides, organic solvents, and other air pollutants [27].

Asthma affects approximately 8% of children in the United States. Asthma disproportionately impacts minority children, especially in lower-income urban communities [28]. More is known about the environmental factors that exacerbate asthma severity than those that cause asthma, but recent evidence has indicated air pollution as a causative factor. Substantial evidence has associated in-utero or early-life exposure to environmental tobacco smoke, ambient and indoor air pollutants, and inhaled allergens (dust mites, pets, and pollens) with asthma incidence and/or severity in children [29,30]. Genetic factors and gene–environment interactions also play a role in asthma causation [31]. Children with specific gene variants were shown to be at increased risk of asthma associated with air pollutants [32]. Environmental exposures may also impact asthma risk through epigenetic mechanisms, an emerging area of study [33,34].

Developmental disabilities (DDs) are associated with lower IQ, learning deficits and other indicators of poor cognitive function, and adverse effects on behavior. One in six children in the United States are affected by DDs [35]. Lower-income children are disproportionally impacted by DDs. Neurotoxicants that have been associated with adverse developmental effects include arsenic, chlorpyrifos, fluoride, lead, manganese, methylmercury, polychlorinated biphenyls, tetrachloroethylene, and toluene [10]. Limited evidence has emerged suggesting an association between exposure to a range of environmental contaminants, including air pollutants, organophosphate pesticides, brominated flame retardants, phthalates, bisphenol A, and perfluorinated compounds, and adverse neurodevelopmental effects [36,37,38,39]. Recent cohort studies in children have suggested a relationship between prenatal exposure to polycyclic aromatic hydrocarbons (PAHs) from air pollution and bisphenol-A with attention problems, anxiety, and aggressive behavior [40,41]. The possible link between environmental contaminants and the increasing prevalence of attention deficit hyperactivity disorder and autism is an area of active investigation [42].

Metabolic syndrome, a cluster of adverse health effects, including obesity, altered lipid levels, and other metabolic abnormalities, is increasing globally. The prevalence of childhood obesity in the US recently stabilized at approximately 18%. Compared with 14% of non-Hispanic White children, 25% of Hispanic and 22% of non-Hispanic Black children are obese [43]. The prevalence of obesity is greater in children with family incomes below the poverty level. There is an increasing body of evidence suggesting that environmental factors may contribute to the rapid increase in the incidence of these metabolic diseases. Evidence that exposure to endocrine-disrupting chemicals can induce effects that manifest later in life as neurological and metabolic outcomes is of particular concern [11,44,45].

### 2.3. Additional Complexity

Multiple susceptibility and vulnerability factors, including developmental stage, behavior, and nonchemical stressors, may modify and/or contribute to the biological response associated with a chemical exposure. Children may be particularly susceptible to health impacts during critical windows of physiologic development [46,47,48]. Children may also be more vulnerable to particular chemical exposures based on behavior, diet, and motor function [49]. Nonchemical stressors may contribute to biological susceptibility or increase potential for exposures. Chemical exposure assessment and risk assessment require population and community-specific information or exposure factors that may vary significantly based on geography and cultural practices. These factors have been reviewed, and a framework was described to facilitate the systematic consideration of these contextual factors for exposure and risk assessment [49,50]. Finally, health outcomes across the lifespan, as well as intergenerational impacts, have been associated with environmental exposures during critical developmental windows [51].

## 3. Conceptual Model of Children’s Environmental Health

**Figure 1:**
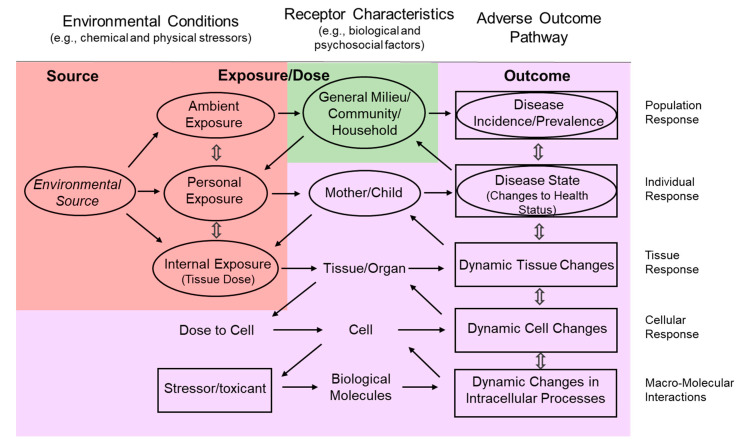
Addressing the Complexity of Children’s Environmental Health Research (adapted from US EPA, 2015).

Given current advances in exposure science, toxicology, and epidemiology, the scope of considerations supporting chemical management decisions that protect children’s health can finally be extended to include a combination of chemical risk and population health perspectives. Traditionally, disparate approaches have been embraced based on discipline-specific experimental systems and measurement technologies. Exposure scientists have focused on understanding the source-to-dose pathways to track chemicals from emission into environmental media to human uptake (source-to-dose), while toxicologists have focused on uptake and subsequent biological activity of the chemical (adverse outcome pathways). Systems thinking enables a conceptual approach for linking information from these disciplines to study the complex interactions between children and environmental stressors (both chemical and nonchemical). Measured indicators across levels of biological organization (molecular to population scale) and across the time course of development can be integrated to characterize this system (Figure 1). Multifactorial exposures to individuals, communities, and populations are captured horizontally from left to right (source-to-dose response with feedback), while the outcome hierarchy is captured vertically from bottom to top (adverse outcome pathway). Though not depicted in this figure, an understanding of the dynamics of these complex systems is required to meet the objective of moving toward development of predictive tools for supporting risk-based decisions [21].

Sources of exposure in the physical environment affect children’s health outcomes through pathways such as those described above. Ellipses indicate source-to-dose pathways while rectangles indicate adverse outcome pathways. The initial flow originates from environmental sources or biological sources, yet the flow is not limited to the forward direction. Left arrows demonstrate the cyclical nature of the exposure pathway. Red, green, and purple shaded sections represent the physical environment, social environment, and child’s health outcomes, respectively.

## 4. A Systems Approach to Evaluate Impacts of Chemicals on Children’s Health

### 4.1. Children’s Environmental Health System Orienters

Systems approaches are critical for understanding and evaluating complex conditions where there are time delays between actions and effects, as well as feedbacks that may result in unintended and difficult-to-predict outcomes. Systems thinking facilitates the awareness of externalities, such as environmental impacts and social costs (health). By invoking systems thinking, the impacts of chemical use on children’s health can be considered to inform socially responsible chemical design, manufacture, and use.

The environmental health of children is influenced by a system of interdependent environmental and social systems, which can collectively be thought of as a complex socioenvironmental system (CSES). In general, to address problems associated with any CSES, we need to elicit and integrate knowledge and assumptions across a range of systems, informing approaches that take into account the complex and uncertain nature of the individual systems and their interdependencies [17].

When managing complex socioenvironmental problems, we need a goal, which can be thought of as a supreme orienter [52], with possible examples including sustainability, resilience, human health, and ecosystem health [17,53]. The supreme orienter captures the essence of the desired goal and may be characterized by several basic orienters. However, the basic orienters are usually abstract and need to be translated into a broad range of concrete operational orienters that can be more easily quantified. These operational orienters are then compared to a range of associated indicators, which are embedded in the individual systems of the CSES. Progress toward achieving the goal is assessed by comparing the operational orienters (the desired state of the CSES) to the associated indicators (the actual state of the CSES) and evaluating the extent of orienter satisfaction. This orienter-based approach provides a flexible and systematic method that can be expanded and adjusted as systems are added to the CSES [17].

**Figure 2:**
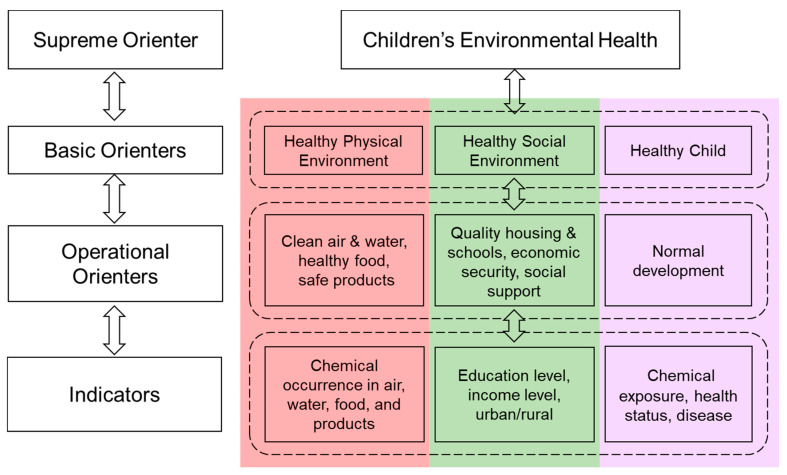
Systems Thinking in Children’s Environmental Health (adapted from Little et al., 2019 [17]).

Thus, children’s environmental health can be assessed by comparing appropriately identified operational orienters to associated indicators or integrated indicators and evaluating the extent of orienter satisfaction, with each orienter requiring a minimum level of satisfaction (Figure 2).

The flowchart on the right corresponds to the systems thinking framework on the left. Each level breaks down across the physical environment, social environment, and child’s health to outline the necessary components of a healthy environment for child development and the relative indicators of environmental health for each of the basic orienters.

The indicators are usually normalized to facilitate weighting and summing. Some orienters and indicators are inherently more important than others, and relative weights may be assigned to account for these differences. The normalized and weighted indicators are summed, resulting in a quantitative description of overall orienter satisfaction. Children’s environmental health is thus considered to be achieved when all operational orienters are satisfied, and may be enhanced by improving individual orienter satisfaction and by achieving the highest possible overall orienter satisfaction [52,54]. The process of deriving operational orienters and selecting suitable indicators, as well as specifying their weights, is guided by stakeholders [55,56]. Because assigning weights requires value judgements [52,54,57], achieving a consensus across stakeholders may be challenging.

For our initial purposes, we considered the desired state for children’s environmental health to be embodied by the following basic orienters: A healthy physical environment, a healthy social environment, and a healthy child or group of children (Figure 2). A healthy physical environment includes clean air, water, healthy food, and safe products. The quality of the social environment is a function of the social and economic resources available to children in their home and community. A healthy child will realize their full potential and develop physically and emotionally within a normal range. Key indicators can be developed and measured to characterize the actual state of these operational orienters, and to evaluate the impacts of environmental health decisions and actions on the orienters and the complex system governing children’s environmental health.

### 4.2. Children’s Environmental Health Indicators

The US EPA defines an indicator as a numerical value derived from actual measurements (or based on estimation methodologies applied to the best available data) of a driver, stressor, state or ambient condition, exposure, or human health or ecological condition. An indicator should answer a specific question, be objective, describe changes, and be comparable across scales [9]. Examples of resources with indicators relevant for evaluating the state of children’s environmental health are highlighted in Table 1.

Integrating disparate data streams and weighting indicators for complex systems in a semiquantitative fashion can inform environmental health decisions across a range of dimensions and perspectives. Tools that enable this integration are becoming increasingly available. One such tool, ToxPi, or Toxicological Prioritization Index (Reif Lab, Raleigh, NC, USA) [63] is an analytical framework that was developed to enable the integration of multiple sources of evidence by transforming data into integrated visual profiles [64]. The platform can be applied to integrate a variety of systems indicators and address a range of children’s environmental health-related questions [65,66,67].

**Figure 3:**
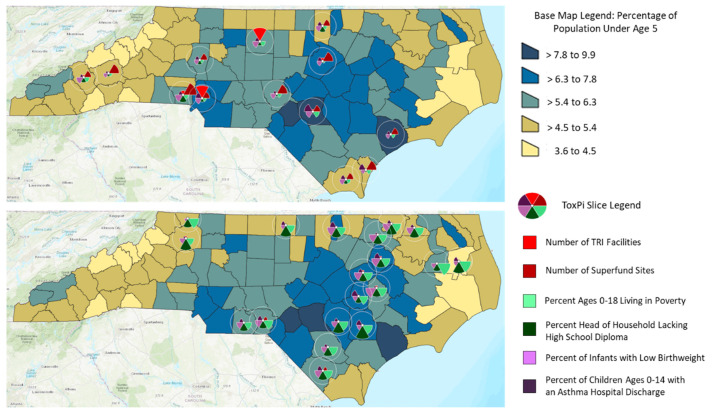
Example of Applying a Systems Approach to Evaluate Children’s Environmental Health in North Carolina.

To demonstrate how a basic systems consideration can be synthesized to enable holistic insights on children’s environmental health, an example of the proposed framework in Figure 3 was implemented using the ToxPi framework. A simple model including indicators of children’s physical environment, social environment, and health status was developed and demonstrated for children in North Carolina using publicly available data. The model included two indicators of children’s health (asthma, low birth weight [68]), two indicators of potential for industrial chemicals in the physical environmental (Toxics Release Inventory (TRI) facilities, Superfund sites [69,70]), and two indicators related to the social environment (income, education [71]). Data were collected at the county level for geographic locations of these known chemical hazard sites, as well as for income, education, and population. ToxPi was used to normalize the data, rank the resulting index, and cluster the counties by K-means analysis into locations with similar properties. The ToxPi charts were then superimposed over a choropleth base map made in ArcGIS (Esri, Redlands, CA, USA) (Figure 3), which shows the percentage of the county population under age five.

In the ToxPi charts, each colored slice represents one of the metrics, and the length of the slice represents the normalized value of that metric, which directly corresponds to the influence on vulnerability. In this example, the counties impacted by the social environment correlate with higher rates of negative health outcomes than those impacted by the physical environment, as visible in the shorter length of the health outcomes (purple) slices in the upper map compared to the lower map (Figure 3).

This information provides insights on the common factors that contribute to vulnerabilities in particular populations. When scaled to include a large number of modifiable variables in each primary bin (health, physical, social), this approach could be valuable for supporting decisions and actions that consider children’s environmental health holistically. In this way, children’s environmental health orienters and indicators can be used to develop hypotheses, guide study designs, and inform decisions to support children’s well-being.

The top map depicts the K-means cluster of counties where negative health outcomes are driven by the physical environment. The lower map depicts the K-means cluster of counties where negative health outcomes are driven by the social environment. 

### 4.3. Implementing a System-Of-Systems Approach for Children’s Environmental Health

The aspirational goal of implementing a system-of-systems approach for children’s environmental health, which integrates complex environmental and social systems and can be used to evaluate actions and predict potential impacts, will require advances in systems modeling platforms and tools [72]. To implement this approach, the use of well-defined models is especially important when addressing societal problems embedded in CSES, because the complexity of such systems overwhelms our ability to understand them [73,74]. This phenomenon, which is sometimes referred to as policy resistance, arises because complex systems are constantly changing, tightly coupled, governed by feedbacks, nonlinear, history-dependent, self-organizing, adaptive and evolving, characterized by tradeoffs, and counterintuitive [74]. As a result, many seemingly obvious solutions to problems fail [74], causing unintended consequences. Models that combine knowledge of mechanistic processes and data-driven insights to automate analyses are thus the primary tools available for understanding complex systems and are essential for adaptive management of interconnected complex systems.

**Figure 4:**
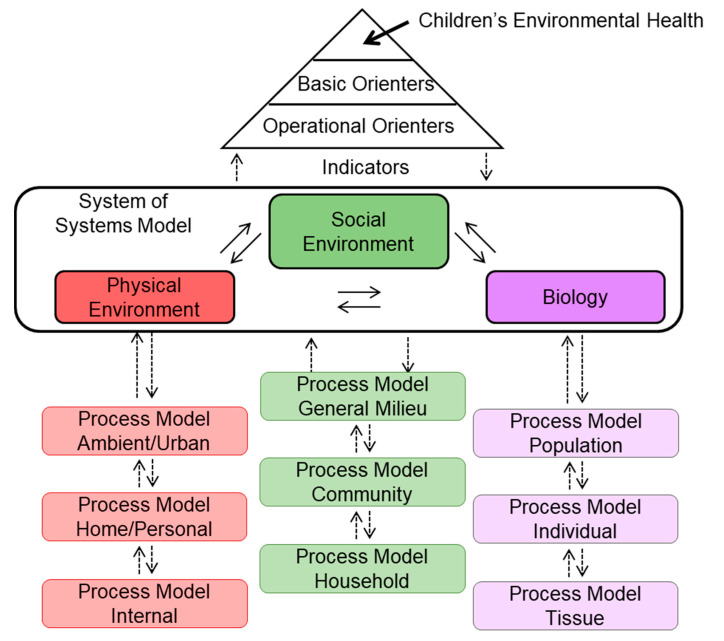
Proposed System-of-Systems Structure for Children’s Environmental Health (Adapted from Little et al., 2019 [17]).

An initial set of systems would need to be defined, as shown in Figure 4. For simplicity, we characterized these as systems representing the physical environment, as well as social systems and biological systems within an individual child. We further characterized the physical and biological systems with models at three scales, including the home environment, local environment, and regional environment for the physical system, and the cellular, tissue, and individual scale for the biological system. The set of basic orienters would be used to derive appropriate operational orienters suitable for these specific systems, as well as to identify associated indicators. These initial systems and indicators would then be used to assess and enhance children’s environmental health. When required indicators to fully describe children’s environmental health are not causally integrated within the available models for the initial systems, they could be connected using a knowledge-based system [75]. Then, depending on their relative importance, they could subsequently be included in additional systems and models that allow these indicators to be causally integrated within the CSES. As new systems are added, additional orienters and indicators could be added that are relevant to the new systems, making the assessment of children’s environmental health increasingly comprehensive and predictive.

Figure 4 depicts the proposed starting model for system-of-systems evaluation of children’s environmental health, separating the distinctions between the social environment, physical environment, and biology while dually emphasizing their innate interconnectedness.

## 5. Conclusions

Systems thinking enables researchers and decisionmakers to integrate the rapidly expanding body of information on children’s environments with advancing insights on child development and health. A systems approach can facilitate the translation of scientific information on key factors across multiple spatial and temporal scales to support decisions that promote and protect children’s health.

The implementation of this approach for any given decision context requires building strength from available information to gain actionable insights. Knowledgebase-driven methods were applied to incorporate information from past and current research. Based on this information, plausible pathways and mechanisms for exposure and toxicity were compiled. Predictive models were developed and applied to estimate conditions under which a chemical will elicit an adverse outcome. Uncertainty was characterized and the predictions were evaluated against the best available benchmarks. In this way, we enabled a holistic interpretation of the available science to build confidence for integration into current risk-based decision paradigms, as well as integration in new ways to evaluate environmental health impacts. This approach can be implemented now by taking advantage of extant data streams, mechanistic models, and aggregating indicators to address complexity. Ultimately, computational platforms will enable rapid linking across the systems and domains that encompass children’s environmental health.

## Figures and Tables

**Table 1:** Examples of Resources for Children’s Environmental Health Indicators.

Resource	Description
Report on the Environment (ROE), US EPA [58]	The ROE shows how the condition of the US environment and human health is changing over time. The purpose of the 80+ ROE indicators is to help answer 23 questions critical to US EPA’s mission of protecting the environment and human health.
America’s Children and the Environment (ACE), US EPA, 3rd edition [59]	The ACE reports data on children’s environmental health. ACE brings together information from a variety of sources to provide national indicators in the following areas: Environments and Contaminants, Biomonitoring, and Health.
EPA CompTox Chemicals Dashboard [60]	A web-based resource for identifying available information on chemicals. It provides access to thousands of chemicals and associated experimental and predicted properties, high-throughput bioactivity data, links to existing Adverse Outcome Pathways (AOPs), and product and functional use data for thousands of chemicals.
CTD (Comparative Toxicogenomics Database) [61]	The CTD is a robust, publicly available database that provides manually curated information about chemical–gene/protein interactions and chemical–disease and gene–disease relationships. These data are integrated with functional and pathway data. The goal is to advance understanding of how environmental exposures impact human health.
EnviroAtlas [62]	EnviroAtlas is a web-based tool that provides geospatial data, integrated indicators, and other resources related to ecosystem services, chemical and nonchemical stressors impacting the environment, and human health.
